# Dynamically Weighted Spatiotemporal Fusion for Deep Learning-Based Prediction of EHA Degradation in Aviation Systems

**DOI:** 10.3390/s26051662

**Published:** 2026-03-06

**Authors:** Tianyuan Guan, Dianrong Gao, Jiangwei Ma, Jing Wu, Yunpeng Yuan, Yun Ji, Jianhua Zhao, Yingna Liang

**Affiliations:** 1 School of Mechanical Engineering, Yanshan University, Qinhuangdao 066004, China; 2 State Key Laboratory of Crane Technology, Yanshan University, Qinhuangdao 066004, China

**Keywords:** EHA, degradation prediction, spatiotemporal characteristic, deep learning, interpretability

## Abstract

Electro-hydrostatic actuators (EHAs) are increasingly deployed in modern aircraft due to their compact size, fast response, and high power-to-weight ratio. However, existing airborne QAR and EICAS data are typically recorded as independent parameters without explicit correspondence to system health states, making degradation assessment and remaining useful life (RUL) prediction challenging. To address this issue, this paper proposes a spatiotemporal degradation modeling framework, termed PreDyn-ST, based on multivariate time series (MTS) data. The method integrates SimCLR-based contrastive pretraining and a dynamic feature fusion mechanism to capture evolving temporal dependencies and spatial sensor correlations. Specifically, graph convolutional networks (GCNs) incorporating physical connectivity priors are employed for spatial modeling, while a Transformer extracts long-range temporal patterns. A learnable dynamic weighting mechanism adaptively balances spatial and temporal features during training. The adaptive behavior is further analyzed using correlation statistical index (CSI) curves for interpretability. Experimental validation on a self-developed EHA degradation test bench and the C-MAPSS benchmark dataset demonstrates that PreDyn-ST achieves competitive and stable prediction performance. In particular, the method shows robust performance under complex operating conditions such as FD004. These results indicate the effectiveness of the proposed framework for accurate and interpretable degradation modeling in aerospace applications.

## 1. Introduction

As the primary power unit of the flight control system, the aircraft hydraulic system provides actuation power for key operations such as takeoff, maneuvering, landing gear retraction, and braking, and is therefore closely associated with flight safety. Currently, most civil aircraft employ centralized hydraulic systems, which suffer from energy losses and transmission inefficiencies. In contrast, Electro-Hydrostatic Actuators (EHAs) offer advantages including compact structure, low weight, rapid response, and high power-to-weight ratio. Due to their integrated direct-drive configuration, EHAs have been gradually introduced as redundant systems in large passenger aircraft and are expected to see broader application in the future [1,2,3]. However, airborne monitoring systems such as QAR and EICAS primarily record independent parameters without directly mapping them to system health conditions. Consequently, degradation assessment relies on indirect inference methods, which limit accurate and real-time health evaluation of hydraulic systems.

Prognostics and Health Management (PHM) focuses on assessing and predicting system health status using sensor data and domain knowledge [4]. Among its core tasks, degradation prediction plays a central role in improving system reliability and reducing maintenance costs [5].

For EHA degradation modeling, three main paradigms are commonly adopted: physical modeling, data-driven modeling, and hybrid approaches [6,7,8]. Due to structural complexity and varying operating conditions, constructing accurate high-fidelity physical models remains challenging in practical applications. As hydraulic systems generate large volumes of operational data, multivariate time series (MTS)-based data-driven modeling has become a practical alternative. Existing data-driven approaches include statistical model-based and machine learning-based methods. For example, Zhang et al. [9] combined the Wiener process with the Ornstein–Uhlenbeck process for life prediction, while Lin et al. [10] developed a nonlinear degradation model with analytical RUL estimation based on the Wiener process.

In contrast, machine learning methods provide stronger feature extraction capability and modeling flexibility compared with traditional statistical approaches. For instance, Ma et al. [11] enhanced EHA degradation prediction by integrating synthetic data generated by TimeGAN with a CNN–BiLSTM–Attention architecture, demonstrating the benefit of deep feature learning under limited data conditions. He et al. [12] combined ARMA regression with a Graph Convolutional Network (GCN) and introduced physical balance constraints into the loss function, thereby improving both predictive accuracy and physical consistency. While these approaches improve modeling capacity, they primarily focus on enhancing feature representation within predefined architectures and do not explicitly consider the evolving interaction between temporal and spatial characteristics during degradation progression.

In recent years, more advanced data-driven RUL prediction approaches have been proposed, including domain adaptation-based methods, federated learning frameworks, and interpretable deep generative models such as variational autoencoders with stochastic degradation formulations. These methods enhance cross-domain robustness, uncertainty quantification, or latent representation interpretability. However, they primarily focus on improving global representation capability or distribution alignment, rather than explicitly modeling the dynamic rebalancing of spatial and temporal feature contributions across different degradation stages. As a result, the stage-dependent evolution of spatiotemporal dominance remains insufficiently explored.

Multivariate time series (MTS) data inherently exhibit spatiotemporal coupling, where temporal dependencies reflect system evolution over time and spatial dependencies arise from structural correlations among sensors. Existing methods largely emphasize temporal modeling using recurrent neural networks (RNNs) [13], convolutional neural networks (CNNs) [14], sequence autoencoders [15], and Transformer architectures [16]. For example, She et al. [17] proposed a BiGRU-based uncertainty estimation framework for interval RUL prediction, while Ong et al. [18] employed a one-dimensional CNN for vibration-based fault diagnosis. Although these models effectively capture temporal patterns, spatial structural relationships are often underutilized. To address this limitation, graph neural networks (GNNs) have been introduced to model inter-sensor dependencies. Wei et al. [19] incorporated temporal convolution and residual connections into a GCN framework to preserve temporal features, Wang et al. [20] proposed a dynamic graph-based structure-aware model (CDSG) for aero-engine RUL prediction, and Li et al. [21] developed a hierarchical attention-based GCN (HAGCN) to jointly capture temporal and spatial dependencies. Nevertheless, most existing approaches adopt static fusion strategies, where spatial and temporal features are combined using fixed structures or weights, without explicitly modeling their dynamic evolution across different degradation stages.

In typical implementations, spatiotemporal features are either concatenated, linearly combined, or fused through fixed attention mechanisms. Although these strategies improve representational richness, the fusion ratio itself remains implicitly fixed after training and does not explicitly reflect degradation-stage-dependent structural variation. Consequently, the evolving dominance between structural correlation and temporal progression is not directly characterized at the modeling level.

Although existing methods have achieved preliminary progress in modeling spatial structures, most of them still suffer from a key limitation: temporal and spatial features are often simply concatenated or superimposed, without considering their dynamic evolution as the system state changes during the degradation process. In particular, the relative importance of spatial and temporal features in system prediction should vary across different stages of degradation. Neglecting this property may prevent the model from accurately capturing system state transitions, thereby reducing its predictive accuracy and adaptability.

In practical engineering scenarios, system degradation leads not only to changes in feature intensity but also to shifts in the relative weighting between spatial and temporal structures. As a result, a fixed fusion strategy is often inadequate for meeting the modeling requirements under complex operating conditions. Therefore, there remains a methodological gap in explicitly modeling the dynamic reallocation of spatiotemporal importance under progressive degradation conditions. To address this challenge, this paper proposes a deep learning approach, PreDyn-ST, capable of dynamically modeling variations in spatiotemporal weights. The objective is to enhance prediction adaptability and improve modeling robustness across different stages of degradation, where distinct types of features may dominate.

The main contributions of this paper are as follows:

To address the dynamic variation in temporal dependence and spatial structure proportion in MTS data caused by different degradation stages, a modeling mechanism based on dynamic weighting is proposed. This mechanism characterizes the evolution of dominant features throughout the degradation process and supports visual analysis of degradation behavior. Moreover, it provides physical interpretability by aligning with known degradation mechanisms, thereby enhancing the transparency and credibility of the model.

A spatiotemporal modeling framework, PreDyn-ST, based on a pre-training strategy, is proposed and implemented. The framework employs SimCLR-based contrastive learning to enhance feature representation within sliding windows, utilizes a Graph Convolutional Network (GCN) and a Transformer to separately model spatial structures and temporal dependencies, and introduces a dynamic weighting mechanism to enable adaptive feature fusion. This supports fine-grained modeling and accurate prediction of the system’s degradation progression.

A custom-built EHA degradation test platform is developed to simulate operational conditions of aerospace hydraulic systems. Real degradation data are collected to conduct modeling and prediction experiments. Experimental results demonstrate that the proposed method not only performs well on the EHA system but also exhibits strong generalization and cross-system adaptability on standard datasets such as C-MAPSS. These results confirm the model’s wide applicability in diverse industrial degradation scenarios.

## 2. Methodology

### 2.1. Feature Enhancement Module: SimCLR-Based Contrastive Learning Mechanism

SimCLR [22] is a contrastive learning method initially developed for visual representation learning and later extended to time series analysis. By leveraging data augmentation, a neural network encoder, and a contrastive loss function, the SimCLR architecture enhances the discriminative power of learned features by increasing inter-sample differences while preserving intra-class similarity, thereby enabling the extraction of informative and distinguishable feature representations. The contrastive learning framework adopted in this study consists of two stages: pre-training and fine-tuning. The pre-training stage employs self-supervised learning without labeled data, whereas the fine-tuning stage utilizes target labels to perform task-specific learning. As a result, despite the use of self-supervision in the initial phase, the overall module is essentially classified under supervised learning.

First, each time series window sample in the dataset is augmented twice using random transformations to generate two related time series windows, ${\tilde{x}}_{i}$ and ${\tilde{x}}_{j}$. Then the encoder network $f\left(\cdot \right)$ is used to extract feature representations from the enhanced time series window and generate feature vectors $h_{i}=f\left({\tilde{x}}_{i}\right)$ and $h_{j}=f\left({\tilde{x}}_{j}\right)$. Finally, the feature vector is passed to the projection head $g\left(\cdot \right)$, which maps the feature representation to the space $z_{i}=g(h_{i})$ and $z_{j}=g\left(h_{j}\right)$ of the application contrast loss. The contrastive loss function employs the normalized temperature-scaled cross-entropy (NT-Xent) loss, which encourages similarity between positive sample pairs while reducing similarity between negative sample pairs. The loss for positive sample pairs is defined as

(1) $$l_{i,j}=-\log\frac{\exp\left(sim\left(z_{i},z_{j}\right)/\tau \right)}{\sum_{k=1}^{2N}1_{\left[k\neq i\right]}\exp\left(sim\left(z_{i},z_{k}\right)/\tau \right)}$$

where $sim(z_{i},z_{j})$ is the cosine similarity between $z_{i}$ and $z_{j}$, defined as $sim\left(z_{i},z_{j}\right)=\frac{z_{i}\cdot z_{j}}{\left\|z_{i}\right\|\cdot \left\|z_{j}\right\|}$; τ is a temperature parameter; $1_{\left[k\neq i\right]}$ is an indicator function, take 1 when k≠i, otherwise 0; The denominator is summed between all 2N possible pairs in the batch, and the positive sample pairs are excluded.

The total loss is obtained by averaging the losses of all positive sample pairs within the batch:

(2) $$L=\frac{1}{2N}\sum_{k=1}^{N}\left(l_{2k-1,2k}+l_{2k,2k-1}\right)$$

It should be noted that adjacent time windows in degradation sequences naturally exhibit high similarity. The contrastive objective does not enforce artificial separation between temporally neighboring samples; instead, it promotes compactness for augmented views of the same window while maintaining relative dispersion at the batch level. Since augmentation magnitude is small and preserves the monotonic degradation trend, the temporal continuity of the degradation process is not disrupted during pretraining.

By minimizing the aforementioned loss functions, the model is able to learn discriminative feature representations that are invariant to data augmentation transformations. These representations preserve sample consistency without altering the fundamental attributes of the data—such as noise injection, scaling, or perturbation—thereby enhancing the model’s generalization capability and robustness to variations in the sample distribution.

**Figure 1:**
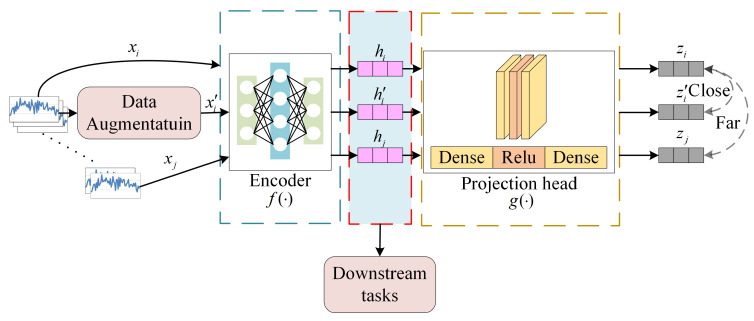
Structure of the SimCLR-based contrastive learning framework. For each sliding window segment, two augmented views are generated and passed through a shared encoder to obtain feature embeddings. A projection head further maps the embeddings into a contrastive space. The contrastive objective maximizes similarity between positive pairs (same window) and minimizes similarity between negative pairs within the batch.

Figure 1 illustrates the structure of a contrastive learning model designed for processing MTS data. The objective of the pre-training stage is to minimize the distance between positive sample pairs while maximizing the distance between negative samples in the feature representation space.

In this study, a widely adopted and effective data augmentation strategy is employed. Specifically, a noise signal $n_{i}$ is randomly sampled from a Gaussian distribution and added to the original sample $x_{i}$, resulting in an augmented version $x_{i}^{\prime }$. Given that samples $x_{i}^{\prime }$ and $x_{i}$ originate from the same raw instance, they are expected to share similar local structures and statistical characteristics and are therefore treated as positive sample pairs. During pre-training, the model reduces the distance between homologous feature representations $x_{i}$ and $x_{i}^{\prime }$, thereby enhancing its ability to recognize shared patterns. Simultaneously, the distance between these representations and non-homologous samples $x_{j}$ is increased, which improves the discriminative capacity of the feature space, reduces computational redundancy, and ultimately enhances prediction accuracy in downstream tasks.

Although contrastive learning does not directly optimize regression loss, it is well-suited for degradation modeling due to the progressive and continuous nature of system health evolution. By enforcing consistency between augmented views of the same sample and separating different samples, the SimCLR objective organizes the embedding space into a locally smooth and stage-consistent manifold that preserves relative degradation ordering. Such structured representation reduces the burden on the downstream regression model, which only needs to learn a smoother mapping from the latent space to the RUL target. Furthermore, the encoder is subsequently fine-tuned under supervised regression loss, enabling task-specific realignment of the feature space and correcting potential discrepancies introduced during pretraining. The Gaussian noise used in augmentation is controlled within a small magnitude and does not alter the intrinsic degradation dynamics; instead, it enhances robustness by encouraging invariance to minor perturbations rather than redefining the degradation trajectory. Therefore, the pretraining stage facilitates, rather than replaces, supervised regression.

The temperature parameter τ controls the sharpness of similarity separation in the contrastive objective. In degradation modeling, τ should maintain a balance between stage discriminability and representation smoothness. In this study, τ = 0.5 provides moderate separation without disrupting the continuous degradation structure, and any potential bias is further adjusted during supervised fine-tuning.

### 2.2. Spatial Modeling Module: Graph Convolutional Network (GCN)

**Figure 2:**
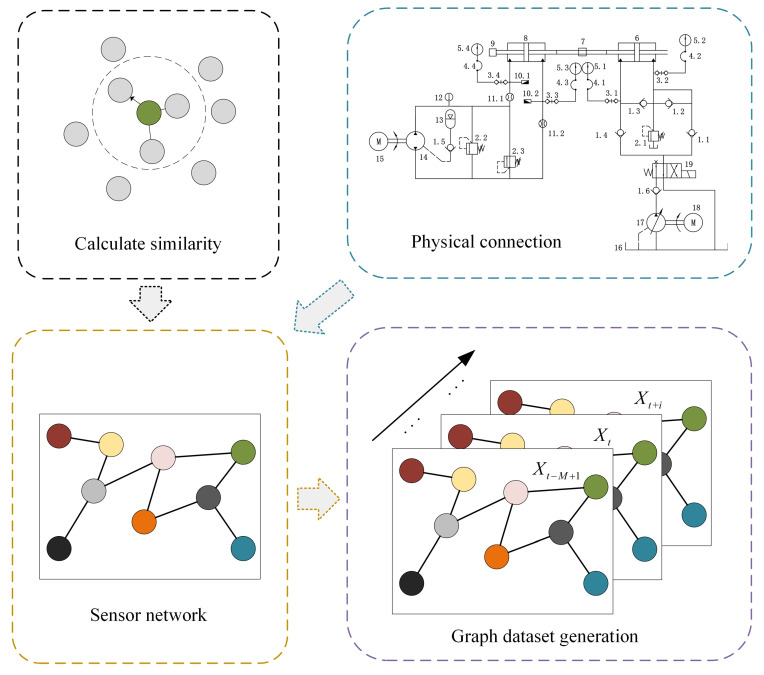
GCN model based on physical prior information.

The Graph Convolutional Network (GCN) [23] is a deep learning model designed for graph-structured data. It effectively extracts spatially dependent features by aggregating information from each node and its neighboring nodes. In the context of multivariate MTS data modeling, GCNs can be employed to capture structural relationships among sensors, thereby enabling the extraction of spatial topological information. Figure 2 illustrates the GCN architecture constructed in this study with the integration of physical prior knowledge.

Specifically, the adjacency structure is first defined according to the EHA system schematic shown in Figure 5. Sensors corresponding to directly connected or functionally coupled components in the hydraulic circuit are linked in the graph based on the structural relationships determined by the system configuration and expert knowledge. On this basis, the edge weights are assigned according to the statistical similarity between sensor signals, which is evaluated through their correlation characteristics. In other words, the physical schematic determines whether a connection exists, while the similarity analysis determines the strength of that connection.

By incorporating both physical connectivity and signal similarity in this manner, the resulting adjacency matrix reflects not only the actual coupling mechanisms within the EHA system but also the data-driven interaction intensity among sensors. This dual consideration enhances the realism of spatial modeling and improves the capability of the network to capture degradation-related spatial correlations.

It should be emphasized that the constructed adjacency matrix serves as a structural prior to constrain information propagation rather than as a dynamic physical simulator. The physical schematic determines the existence of structural connections, while statistical similarity modulates their strength based on signal coupling patterns. The evolution of degradation-related interactions is implicitly captured through feature propagation and parameter optimization during training, instead of through explicit iterative reconstruction of the graph topology. A deeper integration of dynamic physical constraints and adaptive graph learning remains an important direction for future investigation.

The constructed graph is intended to capture statistical and structural dependencies among sensor signals rather than to explicitly model the directional energy flow of the hydraulic circuit.

In Graph $G=\left(V,\xi ,A\right)$ formed by MTS data: V is the number of nodes, that is, the number of sensors; ξ is the edge set between each pair of connected nodes; $A\in {\mathbb{R}}^{N\times N}$ is the adjacency matrix that shows the spatial relationship between nodes; $L=I_{n}-D^{-1/2}AD^{-1/2}$ is a symmetric normalized graph Laplacian matrix, and the diagonal matrix D is calculated according to the adjacency matrix, that is, $D_{i,i}=\sum_{j=1,j\neq i}^{n}A_{i,j}$; $\Lambda ={\left\{\lambda_{i}\right\}}_{i=0}^{n-1}$ is the eigenvalue of L. The corresponding graph convolution operation, the convolution of vertex embedding and filter f is expressed as

(3) $$h=x{*}_{g}f=U\left(\left(U^{T}x\right)⊙\left(U^{T}f\right)\right)$$

where $U={\left[u_{i}\right]}_{i=0}^{n-1}$ is the eigenvector matrix of the Laplacian matrix, and ⊙ is the Hadamard product. In order to further simplify and concretize the process, a diagonal matrix $g_{\theta }$ is introduced, which is the diagonal matrix representation of $U^{T}f$.

(4) $$g_{\theta }=diag(U^{T}f)=diag(\theta_{0},\theta_{1},\ldots ,\theta_{n-1})$$

where diag is the operation of transforming the vector into a diagonal matrix, and $\theta_{i}$ is each feature component projected to the frequency domain. Substituting this formula into the initial graph convolution formula, we can get:

(5) $$h=x{*}_{g}f=Ug_{\theta }U^{T}x$$

This process can be regarded as the process of filtering the graph signal in the frequency domain. Firstly, $U_{T}$ is projected to the frequency domain, then $g_{\theta }$ is used for feature filtering, and finally U is projected back to the time domain, where $g_{\theta }$ is the filter parameter in the frequency domain. In order to reduce the computational complexity and improve the expression ability and stability of the model, the Chebyshev polynomial [24] is introduced to simplify the graph convolution calculation.

(6) $$h=U\left(\sum_{k=0}^{K-1}\theta_{k}^{\prime }T_{k}\left(\tilde{\Lambda }\right)\right)U^{T}x=\sum_{k=0}^{K-1}\theta_{k}^{\prime }T_{k}\left(\tilde{L}\right)x$$

where $\tilde{\Lambda }=2\Lambda /\lambda_{\max}-I_{n}$ and $\tilde{L}=2L/\lambda_{\max}-I_{n}$ are the eigenvalues and Laplacian matrices after re-scaling, $\theta_{k}^{\prime }\in {\mathbb{R}}^{k}$ is the Chebyshev coefficient, $T_{k}\left(\tilde{\Lambda }\right)$ is the Chebyshev polynomial of order k, and $\sum_{k=0}^{K-1}\theta_{k^{\prime }}T_{k}\left(\tilde{L}\right)$ is an explicitly defined convolution kernel.

It should be clarified that the Chebyshev polynomial-based spectral graph convolution is a widely adopted formulation in graph neural network literature, particularly in sensor network and prognostics applications. Its use in this study is motivated primarily by computational efficiency and numerical stability, as it enables localized multi-hop filtering without requiring explicit eigen-decomposition. In practical engineering scenarios, graph convolution is typically employed to model statistical and structural dependencies among sensor signals rather than to explicitly simulate directional physical energy flow. Under such settings, the symmetric normalized Laplacian is commonly adopted to ensure stable propagation and real-valued spectral characteristics. The impact of explicitly incorporating directed graph operators for modeling fluid dynamic directionality remains a potential extension for future work.

In the proposed PreDyn-ST framework, the GCN module is employed to model the spatial structural dependencies among sensors. A dynamic weighting mechanism is introduced to adjust the relative importance of spatial features across different stages of degradation. This adaptive mechanism enhances the system’s ability to model and predict complex degradation behaviors with improved accuracy.

### 2.3. Temporal Modeling Module: Transformer Encoder

The Transformer model [25] is a deep learning architecture that has demonstrated strong performance in time series analysis, primarily due to its attention-based mechanism. Compared with traditional recurrent neural networks (RNNs) and convolutional neural networks (CNNs), the Transformer incorporates a distinctive self-attention mechanism along with positional encoding. This design allows the model to more effectively capture dependencies across different time steps. In particular, the self-attention computation mechanism evaluates the similarity between each time step and all others, enabling the model to dynamically adjust the degree of attention assigned to different temporal positions. The mathematical formulation of this mechanism is as follows:

(7) $$\mathrm{Attention}\left(Q,K,V\right)=\mathrm{softmax}\left(\frac{QK^{T}}{\sqrt{d_{k}}}\right)V$$

where Q, K, V is the query, key and value matrix respectively, and $\mathrm{softmax}\left(\frac{QK^{T}}{\sqrt{d_{k}}}\right)$ calculates the similarity between each element in the sequence and other elements, so as to determine the weight of its attention.

This mechanism enables the model to flexibly attend to different parts of the sequence at each time step, without being constrained by fixed windows or distances, thus effectively capturing long-term dependencies. Simultaneously, the multi-head attention mechanism is implemented within the Transformer to further enhance the capability of self-attention. By computing multiple self-attentions in parallel, the model can focus on information from different subspaces. The mathematical expression of the multi-head attention function is

(8) $$\mathrm{MultiHead}\left(Q,K,V\right)=\mathrm{Concat}\left({\mathrm{head}}_{1},{\mathrm{head}}_{2},\ldots ,{\mathrm{head}}_{h}\right)W^{O}$$

where each attention head is calculated as follows:

(9) $${\mathrm{head}}_{i}=\mathrm{Attention}\left(QW_{i}^{Q},KW_{i}^{K},VW_{i}^{V}\right)$$

This mechanism allows the model to analyze sequence correlations from multiple perspectives at each time step, thereby enhancing its capability to capture and extract temporal dependencies.

Since the Transformer does not inherently possess sequential information, positional encoding introduces unique representations for each position by utilizing sine and cosine functions, thereby preserving the sequence’s positional information. The formula is

(10) $${\mathrm{PE}}_{\left(pos,2i\right)}=\sin\left(\frac{pos}{10000^{2i/d_{\mathrm{model}}}}\right)$$

(11) $${\mathrm{PE}}_{\left(pos,2i+1\right)}=\cos\left(\frac{pos}{10000^{2i/d_{\mathrm{model}}}}\right)$$

where pos is the location index, i is the dimension index, and $d_{\mathrm{model}}$ is the dimension of the model. The introduction of positional encoding enables the model to capture the sequential relationships among time steps in a time series, thereby preserving the global temporal structure of the data.

It should be noted that the sinusoidal positional encoding adopted in this study provides relative ordering information within each input window rather than imposing any periodic or monotonic constraint on the degradation process. Although the sliding-window sampling strategy introduces a nonlinear mapping between position index and physical time, the encoding primarily preserves local temporal ordering. The periodic form of sinusoidal functions does not enforce cyclic degradation behavior; instead, it offers a smooth positional representation to facilitate attention-based dependency modeling. Furthermore, the multi-head attention mechanism allows different subspaces to capture short-term fluctuations and long-term degradation trends adaptively, enabling multi-scale temporal representation learning.

**Figure 3:**
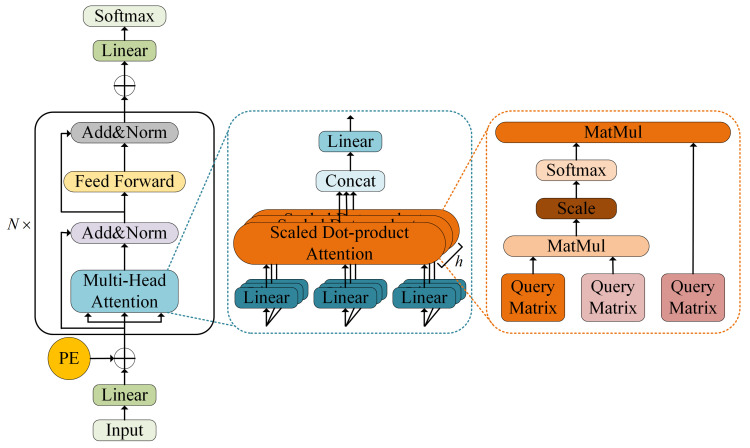
Model structure of Transformer.

Figure 3 illustrates the architecture of the Transformer module. In the proposed model, the Transformer encoder is utilized to capture temporal dependencies within the multi-sensor degradation sequences. This component is capable of identifying long-range correlation patterns, thereby improving the accuracy of degradation trend modeling. When combined with the subsequent dynamic weighting mechanism, the module can flexibly adjust the contribution of temporal information according to different stages of degradation, enabling adaptive modeling of spatiotemporal characteristics.

### 2.4. Overall Model Architecture: PreDyn-ST Spatiotemporal Modeling Process

**Figure 4:**
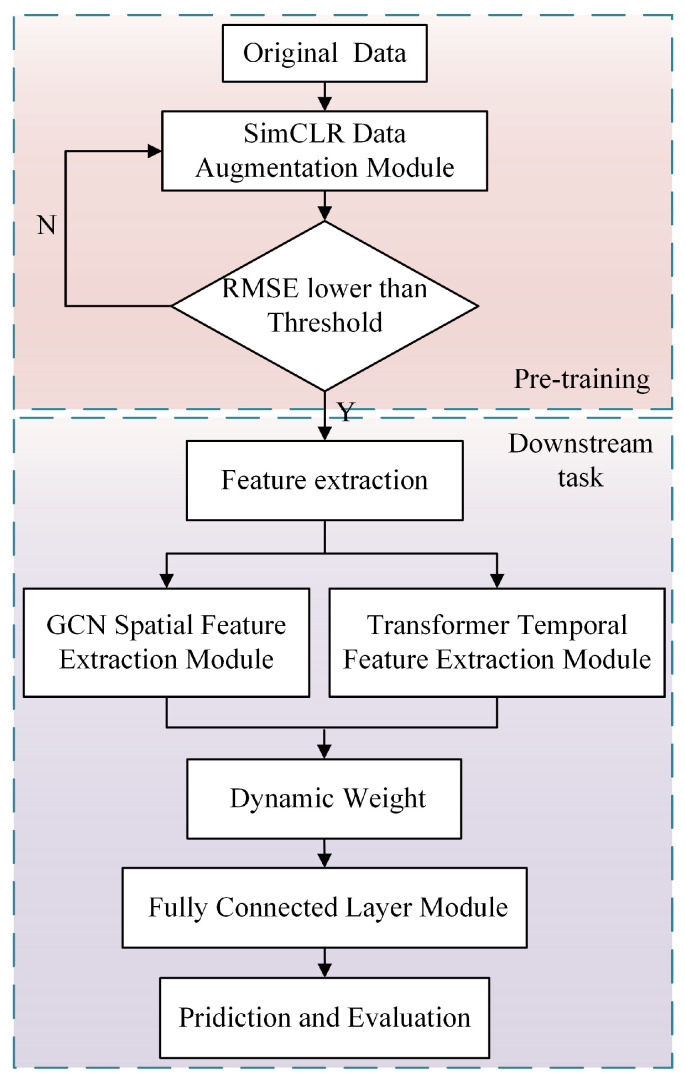
Framework flowchart of PreDyn-ST.

Figure 4 illustrates the overall framework of the proposed PreDyn-ST model, which adopts a conventional two-stage modeling strategy consisting of pre-training and fine-tuning. The entire process is divided into two stages: the pre-training stage focuses on feature enhancement and initialization, while the downstream stage performs feature extraction, dynamic modeling, and RUL prediction.

In the pre-training stage, the original multivariate time series (MTS) data are processed using SimCLR-based contrastive learning to increase the similarity between positive sample pairs and enhance their distinction from negative ones. These sample pairs are generated through augmentation techniques such as Gaussian noise injection and amplitude transformation. The encoder is optimized using the NT-Xent loss minimization strategy. Once the enhanced data reach a predefined root mean square error (RMSE) convergence threshold during training, the pre-training process is terminated, and the encoder output is employed to initialize the downstream task.

In the downstream stage, the pre-trained feature representations are first passed through a basic feature extraction layer to obtain a unified embedding format. These representations are then fed in parallel into two submodules: the Transformer-based temporal modeling module and the GCN-based spatial modeling module. The Transformer module captures temporal dependencies within the input sequence using a multi-head attention mechanism, which facilitates the modeling of long-range correlations. In parallel, the GCN module learns the spatial structural relationships among sensors through graph convolution operations, thereby extracting the system’s topological dependency information. The outputs of the two modules represent the temporal features and spatial embeddings of the degradation sequence, respectively.

To address the dynamic variation in the importance of temporal features and spatial structures across different stages of system degradation, this study introduces a dynamic weighting mechanism for feature fusion. During the training process, the model automatically learns a set of updateable fusion weight parameters that adapt to the evolving correlations among sensors throughout the degradation process. This mechanism enhances the model’s sensitivity to dominant feature structures during degradation and improves the overall robustness of the modeling process.

It is important to distinguish this dynamic weighting strategy from conventional adaptive gating mechanisms such as those in LSTM networks. Unlike element-wise gating functions that regulate recurrent state transitions, the proposed mechanism operates at the module-fusion level by learning a scalar coefficient that balances spatial and temporal representations. During training, this coefficient is jointly optimized via backpropagation, proportionally redistributing gradient flow between the two branches. When one branch exhibits reduced discriminative capacity, the loss gradient naturally adjusts the fusion weight to emphasize the more informative pathway. Since the fusion parameter is low-dimensional, the optimization landscape remains simple and does not introduce significant risk of local optima, while enabling stable adaptive rebalancing between spatial smoothing and temporal dynamics.

As noted in reference [12], the degradation process in real-world systems is often nonlinear. With increasing degradation severity, inter-sensor correlations tend to strengthen, and the spatial structure progressively converges. Taking the EHA system as an example, hierarchical failure pathways—such as fluid–solid coupling and contaminant propagation—may occur. This mechanism results in a gradual reduction in the spatial structural differences among sensors. Consequently, the dynamic weighting mechanism plays a critical role in modeling system-level degradation trends.

Finally, the fused spatiotemporal features are passed through a fully connected layer module. This module employs a multi-layer perceptron to progressively compress the feature dimensionality and project the high-dimensional spatiotemporal representation into the target space for remaining useful life prediction, thereby generating the final degradation output.

## 3. Degradation Prediction of the EHA System

### 3.1. Overview of the EHA System

**Figure 5:**
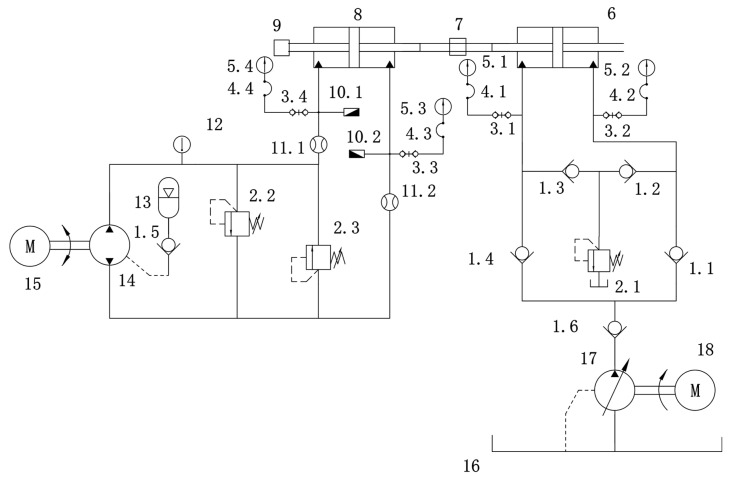
Hydraulic schematic diagram of EHA performance degradation test bench.

Figure 5 presents a schematic diagram of the hydraulic system used in the EHA performance degradation test bench constructed in this study. The test platform is designed to simulate the operating conditions and performance degradation processes of typical actuators within flight control systems. It comprises two main components: an active EHA subsystem and a passive load subsystem.

It is specifically composed of the following components: one-way valve 1, pressure relief valves with attached directional spool valve 2, pressure measuring joint 3, pressure measuring hose 4, shockproof pressure gauge 5, hydraulic cylinder 6, force sensor 7, hydraulic servo cylinder 8, displacement sensor 9, pressure sensor 10, flow sensor 11, temperature sensor 12, accumulator 13, bidirectional quantitative gear pump 14, AC servo motor 15, oil tank 16, one-way variable gear pump 17, three-phase asynchronous motor 18, two-position four-way electromagnetic directional valve 19.

The experimental platform supports controllable testing conditions, including operating mode adjustment, load disturbance, and contamination simulation. It facilitates a range of tasks such as degradation process modeling, life prediction validation, and interpretability analysis. In the subsequent modeling section, multi-channel time series data collected from this platform will be used to evaluate the modeling and prediction capabilities of the proposed PreDyn-ST method under real system conditions.

### 3.2. Fluid–Solid Coupling-Induced Failure in Hydraulic Systems

In hydraulic systems, flow pulsation induced by gear pump operation is a major contributor to system degradation. This pulsation, when combined with factors such as damping, inertia, and throttling losses, leads to periodic fluctuations in both fluid pressure and velocity. As the disturbance propagates through the structure, it excites frequency responses across multiple pipeline segments and connection nodes. When the excitation frequency approaches the system’s natural frequency, it can trigger amplification effects and cause mechanical fatigue damage [26,27,28].

This phenomenon reflects a cascade effect during the degradation process, where localized disturbances propagate through hierarchical structures and intensify progressively. To investigate this mechanism, the present study constructs a dynamic modeling framework for the hydraulic system from the perspective of fluid–solid coupling.

The periodic flow pulsation is represented using a Fourier series expansion, and the instantaneous flow rate can be expressed as

(12) $$Q\left(t\right)=\frac{a_{0}}{2}+\sum_{i=1}^{l}\left(a_{i}\cos\left(i\omega t\right)+b_{i}\sin\left(i\omega t\right)\right)$$

In the formula, $\omega =\frac{2\pi }{T}$, $a_{0}=\frac{2}{T}\int_{0}^{T}Q\left(t\right)dt$, $a_{i}=\frac{2}{T}\int_{0}^{T}Q\left(t\right)\cos\left(i\omega t\right)dt$, $a_{i}=\frac{2}{T}\int_{0}^{T}Q\left(t\right)\cos\left(i\omega t\right)dt$, $b_{i}=\frac{2}{T}\int_{0}^{T}Q(t)\sin(i\omega t)dt$, T is the flow pulsation period, and I is the number of observable harmonics.

Combine the same frequency terms in the formula to get another form:

(13) $$Q\left(t\right)\approx A_{0}+\sum_{i=1}^{l}A_{i}\sin\left(i\omega t+\phi_{i}\right)$$

In the formula, $A\;=\;\frac{a_{0}}{2}$, $A_{i}=\sqrt{a_{i}^{2}+b_{i}^{2}}$, $\phi_{i}=\arctan\left(\frac{b_{i}}{a_{i}}\right)$.

To construct a structural response modeling framework, the hydraulic pipeline is treated as a continuous medium system consisting of elastic, viscous, and inertial components. The transfer matrix method (TMM) is employed to model the dynamic coupling relationships among individual pipeline segments. Its fundamental formulation is given by:

(14) $$\phi_{i}^{R}=M\phi_{i}^{L}$$

where M is the transfer matrix of the pipe segment i, and ϕ represents the state variable vector at a given position.

For discontinuous connection points or pipe support structures, a support constraint matrix N is introduced to represent the associated boundary conditions:

(15) $$\phi_{i+1}^{L}=N\phi_{i}^{R}$$

Based on the above modeling, the cascade propagation and frequency-domain response of fluid excitation within the pipeline system can be simulated. This provides both the physical foundation and structural basis for subsequent spatiotemporal feature modeling and degradation path identification.

### 3.3. Contaminant Propagation-Induced Failure in Hydraulic Systems

In hydraulic systems, prolonged operation of frictional pairs results in significant particle wear. This is especially evident in high-pressure contact regions such as valve spools, pistons, and cylinders, where wear particles are entrained in the hydraulic fluid and gradually accumulate at pipeline junctions and component interfaces. This contaminant accumulation is one of the key physical mechanisms responsible for the performance degradation and eventual failure of hydraulic systems [29,30,31]. Previous studies have shown that particle contamination not only decreases the viscosity of the working fluid and accelerates wear but also leads to a decline in sensor signal quality and instability in feedback control systems. To quantitatively describe the contaminant accumulation process, this study introduces a differential model for pollution concentration dynamics. Let $N_{0}\left(t\right)$ denote the concentration of abrasive particles in the tank, i represent a component selected from the pump, pipeline, valve, or cylinder, and $R_{i}\left(t\right)$ denote the pollutant generation rate due to wear. The pollution balance equation for a component $\left[t,t+\Delta t\right]$ at any given time is expressed as

(16) $$\frac{dN_{i}\left(t\right)}{dt}=E_{v}\left(t\right)Q-R_{v}\left(t\right)$$

In the formula, $E_{v}\left(t\right)$ is the equivalent filtration efficiency and Q is the flow rate.

The two aforementioned cascade effects propagate progressively through the power, control, and actuator subsystems, each being manifested at different hydraulic components. These effects jointly accelerate system degradation. Combined with the earlier observation that sensor correlations within the system tend to increase as degradation progresses, it can be inferred that the initial failure of the EHA system arises from the coexistence of multiple degradation modes across different components. As degradation deepens, the cascade effect intensifies and spreads throughout the system, leading to convergence in failure patterns across components. Consequently, sensor signals from different components of the EHA system begin to exhibit increasing similarity, reflected in higher inter-sensor correlation and diminished spatial differentiation.

As degradation continues, contaminant accumulation further promotes convergence in the degradation trends of various components. This, in turn, reduces the variability in the output characteristics of multi-channel sensor data, as evidenced by increased signal correlation and reduced spatial heterogeneity. This cascade process—from contamination to degradation to signal homogenization—exacerbates the challenges of spatiotemporal decoupling and multi-modal analysis in degradation modeling. Therefore, the proposed contaminant propagation model not only captures the latent physical degradation mechanisms within the system but also provides a theoretical foundation for the dynamic weighting mechanism employed in the subsequent spatiotemporal feature fusion process.

### 3.4. Overall Framework for EHA Performance Degradation Prediction

**Figure 6:**
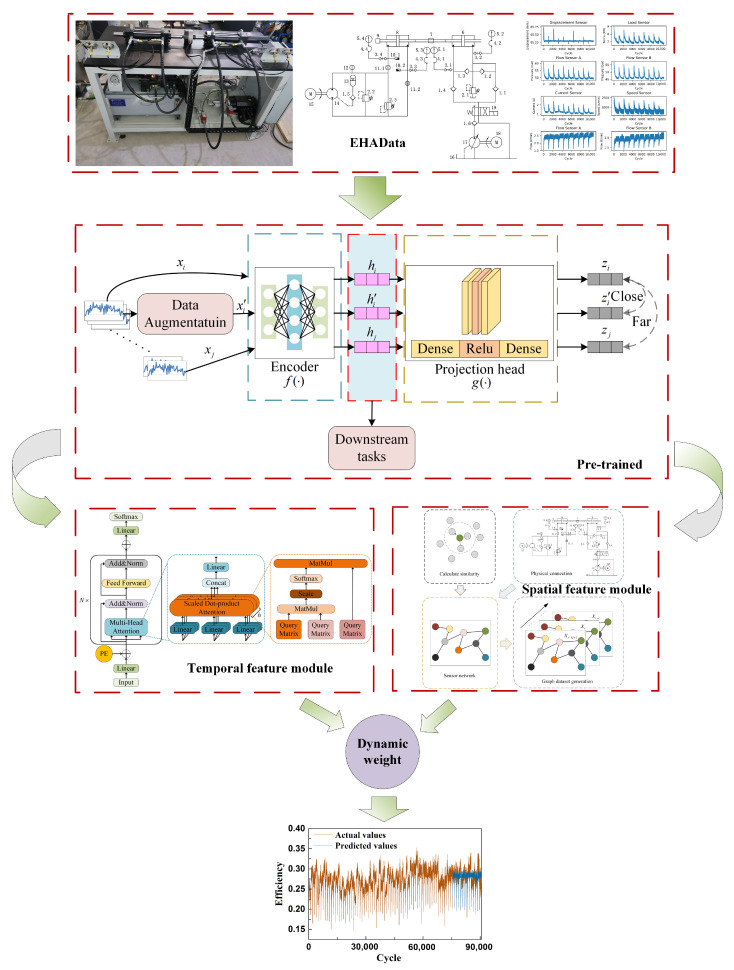
Overall framework for predicting EHA performance degradation.

Figure 6 illustrates the complete implementation process of the proposed PreDyn-ST method for EHA performance degradation modeling. The workflow begins with multi-channel raw signals collected from the experimental platform and proceeds through several stages, including data preprocessing, feature modeling, dynamic fusion, and life prediction.

In the data preprocessing stage, the collected sensor signals are segmented using a sliding window approach to construct MTS samples. During the pre-training stage, the SimCLR framework is employed to perform contrastive learning in order to enhance the model’s ability to characterize degradation patterns. By constructing positive and negative sample pairs, the encoder is guided to generate discriminative representations of the time series.

In the downstream task modeling stage, the encoder outputs are fed into two parallel branches: a Transformer module for extracting temporal dependencies and a GCN module for capturing spatial structural features. This step establishes the joint modeling capacity for dynamic behaviors and topological coupling among sensors in the EHA system.

To accommodate the evolving dominance of temporal and spatial characteristics throughout the degradation process, a dynamic weighting mechanism is introduced. Specifically, the fusion weights are implemented as learnable parameters that are jointly optimized with the entire network through backpropagation by minimizing the overall regression loss. During training, the model automatically adjusts the contribution of spatial and temporal features according to the data-driven optimization process, rather than relying on manually predefined rules or external statistical indicators. Finally, the degradation state or RUL at each time point is predicted through a fully connected regression module.

### 3.5. Data Description and Preprocessing

**Figure 7:**
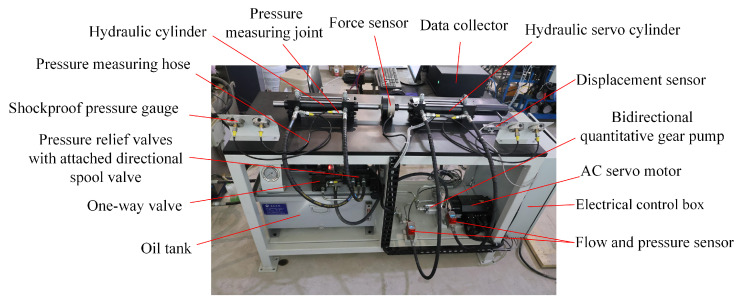
EHA performance degradation test bench.

Figure 7 shows the EHA performance degradation test bench. The platform consists of two main sections: an active unit driven by the EHA and a hydraulic load unit. The active part includes a servo motor, gear pump, servo hydraulic cylinder, and multiple sensors. The load section adopts an opposing-cylinder structure and is composed of a motor pump, a relief valve, and several one-way valves.

During operation of the EHA system, the high-speed servo motor drives the gear pump to convert electrical energy into hydraulic energy, which is then delivered to the piston chamber of the servo cylinder to generate thrust and induce reciprocating motion of the piston. When the control system issues a target displacement or velocity command, the motor regulates the output flow and pressure of the pump to realize controlled piston motion. On the load side, the system pressure is adjusted via a relief valve to simulate varying load magnitudes. All sensor signals are synchronously collected through a custom acquisition program using a data acquisition card.

**Figure 8:**
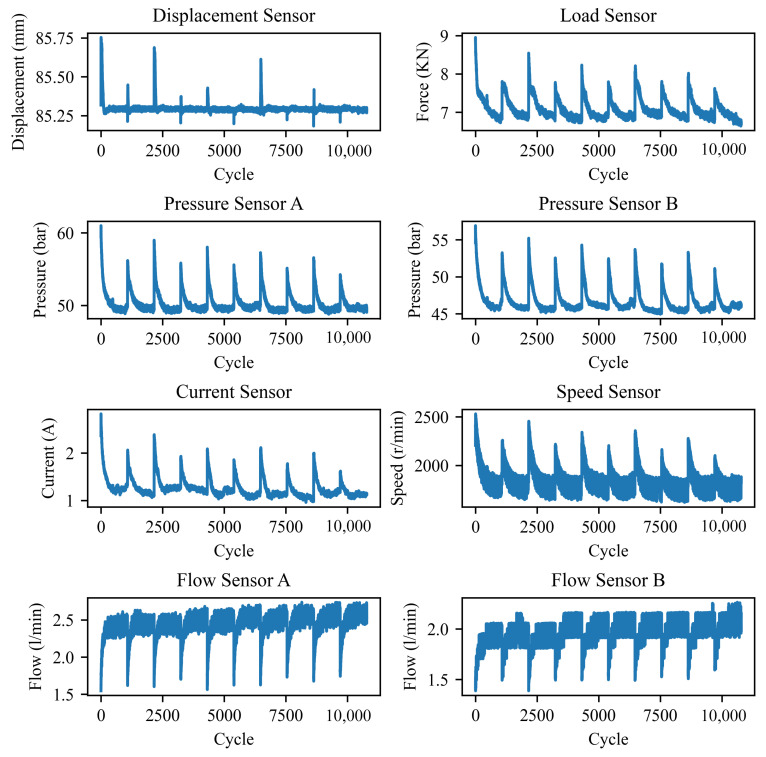
Sensor signal of EHA.

A total of eight types of sensor signals were recorded during the experiment. Figure 8 displays the data collected over the first ten days, including piston rod displacement, load force (measured by a force sensor), and flow and pressure signals from both chambers of the hydraulic servo cylinder, as well as the current and rotational speed of the servo motor. The experiment employed sinusoidal excitation, where the servo cylinder executed reciprocating motion at a frequency of 1 Hz with a stroke length of 30 cm. The sampling frequency was set to 1000 Hz, and data acquisition was performed for three hours per day over a period of 84 days, thereby capturing the system’s full dynamic response throughout its entire life cycle.

As the displacement signal exhibited minimal variation during the degradation process, it was excluded from the model input. Consequently, only the remaining seven sensor signals were used for modeling. In total, 906,360 periodic samples were collected, with each cycle comprising 1000 time steps. To reduce computational cost and memory usage, a sliding window down sampling strategy was applied, where the maximum value from every 10 cycles was extracted and used as the final input feature.

For label construction, system efficiency is adopted as the degradation indicator. It is defined as the ratio between the displacement response and the corresponding unit control input. The efficiency values are computed from the raw experimental data using a custom Python 3.6.8 script. To ensure temporal alignment with the input features, the average system efficiency over every 10 cycles is calculated and used as the corresponding prediction label.

Accordingly, the graph structure for the EHA dataset is constructed using these seven sensors, resulting in a 7 × 7 adjacency matrix.

### 3.6. Model Training Process

Based on Python and the PyTorch deep learning framework, this study constructs the PreDyn-ST model, which integrates SimCLR, GCN, Transformer, and a dynamic fusion mechanism. To perform degradation modeling and validation, the EHA dataset collected over the 84-day life cycle is partitioned proportionally: the first 70% is used for training and validation, while the remaining 30% is reserved for testing.

The training process consists of two stages. In the first stage, SimCLR pre-training is conducted to enhance the model’s ability to represent temporal features. The corresponding network architecture and parameter settings are provided in Table 1. In the second stage, downstream modeling is performed, including GCN-based spatial modeling and Transformer-based temporal modeling. Key structural parameters for these modules are summarized in Table 2.

**Figure 9:**
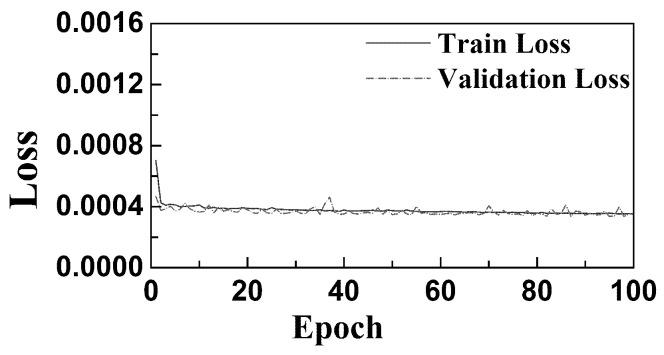
Training and validation loss of EHA data.

Figure 9 presents the training and validation loss curves on the EHA dataset. It can be observed that, after contrastive learning-based pretraining, both training and validation losses decrease rapidly. The model reaches convergence within a relatively small number of epochs, indicating that the introduction of contrastive pretraining significantly accelerates convergence. Moreover, the model demonstrates good fitting ability to both training and validation data.

### 3.7. Evaluation Index

To enable a fair comparison between the proposed model and other mainstream RUL prediction methods, and following prior studies [14,21,32,33,34,35], this study adopts three widely used regression evaluation metrics: mean absolute error (MAE), root mean square error (RMSE), and a score function (Score). These metrics evaluate prediction performance from three perspectives: average deviation, error fluctuation, and robustness-weighted accuracy.

Specifically, MAE quantifies the average absolute difference between the predicted values and the ground truth, as defined in Equation (17), and reflects the overall prediction deviation. RMSE, defined in Equation (18), emphasizes error variability and is particularly sensitive to uneven error distributions.

Given that the target system in this study is an EHA system with system efficiency values typically close to 1, the Score index is less responsive under such conditions. Therefore, MAE and RMSE are adopted as the primary evaluation metrics for error quantification.

In addition, to account for the asymmetric risk between overestimation (i.e., predicted life exceeds actual life) and underestimation, a weighted Score function, commonly used in C-MAPSS evaluations, is introduced (see Equation (19)). This function penalizes over-prediction errors more heavily, thereby providing a more balanced assessment of model robustness and fault tolerance from a safety-critical perspective.

(17) $$\mathrm{MAE}=\frac{1}{n}\sum_{i=1}^{n}\left|x_{i}-x_{ip}\right|$$

(18) $$RMSE=\sqrt{\frac{1}{n}\sum_{i}^{n}{\left(x_{i}-x_{ip}\right)}^{2}}$$

(19) $$d_{i}={\hat{y}}_{i}-y_{i},\;Score=\sum_{i=1}^{N}s_{i},\;s_{i}=\left\{\begin{array}{ll} \exp\left(-\frac{d_{i}}{13}\right)-1, & d_{i}<0\\ \exp\left(\frac{d_{i}}{10}\right)-1, & d_{i}\geq 0 \end{array}\right.$$

where n represents the total number of samples to be evaluated, $x_{i}$ represents the true RUL, the prediction error is defined as $d_{i}={\hat{y}}_{i}-y_{i}$.

### 3.8. Results Analysis

**Figure 10:**
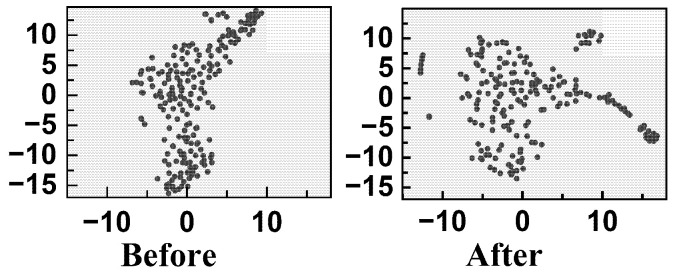
Feature distribution of EHA data before and after pretraining.

To evaluate the contribution of each component to the overall model performance, ablation experiments were conducted on the EHA dataset, as summarized in Table 3. The comparison models include: a baseline GCN model, a baseline Transformer model, pre-trained versions of both the GCN and Transformer, a GCN–Transformer architecture with a dynamic weighting mechanism, and the full PreDyn-ST model, which integrates both contrastive pretraining and dynamic spatiotemporal fusion. The results demonstrate that contrastive pretraining consistently improves model performance. For example, the RMSE of the baseline GCN model decreases from 2.15 to 1.93, while that of the Transformer model decreases from 1.93 to 1.89, indicating that contrastive learning enhances the initial feature representation capability. Furthermore, incorporating the dynamic weighting mechanism enables the GCN–Transformer structure to better capture evolving spatiotemporal dependencies during the degradation process, resulting in improved prediction accuracy and adaptability. Ultimately, the complete PreDyn-ST framework achieves the best performance across both RMSE and MAE metrics (1.86 and 0.0148, respectively). To further verify that the observed improvement is not attributed to random initialization, five independent runs were conducted for both the pre-trained Transformer and PreDyn-ST under identical settings. The pre-trained Transformer achieved an RMSE of 1.890 ± 0.016, while PreDyn-ST achieved 1.860 ± 0.009. A paired t-test on RMSE across runs yielded *p* < 0.001, indicating that the performance gain introduced by the dynamic weighting mechanism is statistically significant. These results confirm that the improvement is consistent and robust rather than incidental. Its dynamic weighting strategy allows the model to automatically adjust the spatiotemporal fusion ratio according to the degradation stage, thereby enhancing both interpretability and generalization across different degradation patterns.

t-SNE is a widely used dimensionality reduction and visualization technique that projects high-dimensional feature representations into a low-dimensional space to reveal their structural organization [36]. Figure 10 shows the two-dimensional projection of 200 EHA samples before and after contrastive pretraining. Compared with the distribution prior to pretraining, the representations obtained after contrastive learning exhibit a more progressively organized spatial arrangement along the degradation trajectory. The embedding space becomes more structurally coherent, with improved distinguishability between different temporal stages of system degradation. These results indicate that contrastive learning enhances the representation consistency and progression awareness in the latent space, which is beneficial for regression-oriented lifetime prediction rather than discrete categorical classification.

During equipment operation, the coupling effect among system submodules typically intensifies as degradation progresses. To quantitatively characterize the degree of correlation among multiple sensors, this study introduces the Correlation Statistical Index (CSI), as defined in Equation (20). CSI is computed by taking the average absolute value of the Pearson correlation coefficients across all sensor pairs, thereby providing a quantitative measure of the overall signal coupling strength within the system [37]. A higher CSI value indicates a stronger correlation among subsystem signals, reflecting an increased level of interdependence as degradation advances.

(20) $$S_{t}=\frac{1}{n^{2}}\sum_{i}\sum_{j}\left|s_{ij}\right|$$

where $s_{ij}$ is the Pearson correlation coefficient between signal i and j, n is the total number of signals.

**Figure 11:**
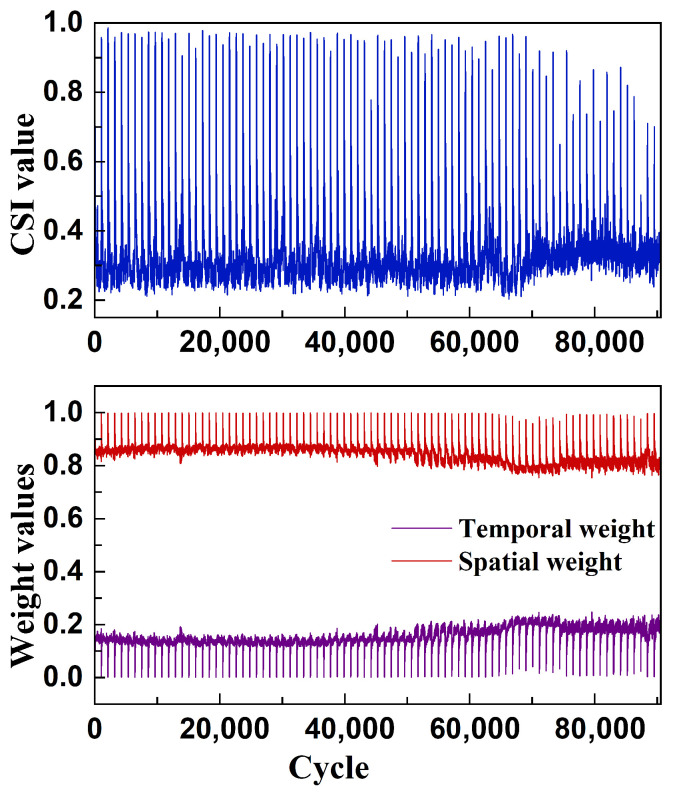
CSI curve and dynamic weight change graph of EHA data.

Figure 11 illustrates the variation trend of the Correlation Statistical Index (CSI) and the corresponding spatiotemporal weight curves throughout the entire operational lifecycle of the EHA system. The results show that in the initial operational phase (0–70,000 cycles), the CSI remains relatively stable, indicating a consistent and undisturbed system state. As the operation continues, CSI exhibits a gradual upward trend, reflecting an increase in inter-sensor correlation and the onset of system degradation. This evolution is consistent with the characteristics of contaminant propagation and fluid–solid coupling failure mechanisms, which tend to exhibit stronger cascading and diffusion effects in the later stages, impacting multiple subsystems simultaneously.

The figure also presents the dynamic weight variation curves of the spatial and temporal modules within the model. Overall, the spatial module maintains dominant importance; however, its weight slightly decreases during the phase in which CSI begins to rise. In contrast, the temporal module gains greater weight during this period. This shift indicates that in the early degradation phase, the system exhibits pronounced structural differentiation, allowing the GCN to effectively capture topological relationships among sensors through the graph structure. In the later stages of degradation, as sensor signals become increasingly similar, the discriminative power of GCN node features weakens, reducing the effectiveness of spatial modeling. At this point, the Transformer module becomes more effective in capturing the intrinsic temporal evolution of the degradation sequence and therefore receives greater emphasis in the model.

When contaminant propagation intensifies, inter-sensor correlation increases, leading to higher similarity among node features in the graph representation. Since the GCN performs neighborhood aggregation through normalized adjacency propagation, increased feature similarity across connected nodes results in reduced embedding variance and enhanced smoothing effects. This effectively weakens spatial discriminability in the latent space. During training, the dynamic weighting mechanism adjusts the fusion ratio through backpropagation, allocating greater emphasis to the temporal branch when spatial discriminability decreases. Therefore, the weight evolution reflects the adaptive rebalancing between spatial smoothing and temporal dynamics rather than being directly driven by CSI.

These observations suggest that the model can adaptively adjust the relative importance of spatial and temporal modeling pathways based on the coupling structure and dynamic characteristics of the input data. This enables the model to respond dynamically to structural sensitivity across different stages of system degradation.

**Figure 12:**
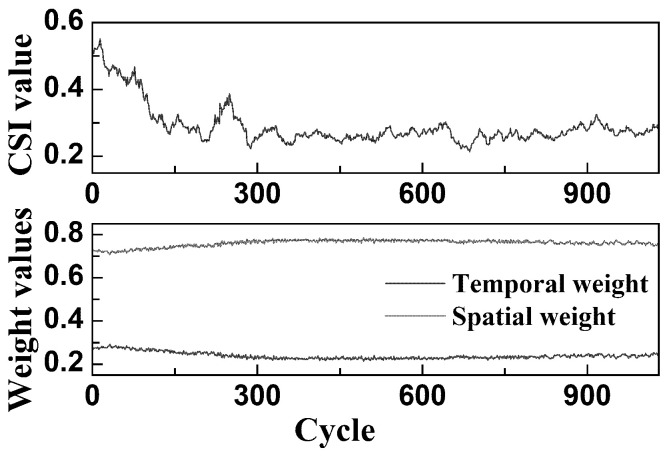
Experimental CSI curve and dynamic weight change curves within one day.

Figure 12 illustrates the variation trends of the Correlation Statistical Index (CSI) and the corresponding model weights over a single-day operational cycle of the EHA system. The CSI curve shows a high value at the beginning of the cycle, followed by a rapid decrease to approximately 0.25, after which it stabilizes with only minor fluctuations. Correspondingly, the weight of the spatial module gradually increases, while the weight assigned to the temporal module decreases. Once the CSI stabilizes, the weights of both modules also become synchronized and steady.

This behavior can be attributed to the evolution of hydraulic system working conditions. During the cold-start phase, the hydraulic oil exhibits high viscosity and poor lubrication performance, resulting in pronounced initial wear. This leads to stronger correlations between sensor signals and an elevated CSI value. As the system continues to operate, the increasing oil temperature reduces viscosity and improves lubrication, allowing the system to transition into a stable operating condition. This change weakens sensor correlations and causes the CSI to decline steadily.

In the modeling process, the variation in inter-sensor correlation directly influences the spatial information propagation capacity of the GCN module. During the early wear phase, high signal correlation results in low node variability within the graph, thereby limiting feature aggregation and reducing the spatial module’s contribution. Once the system enters a stable operating state, greater signal differentiation enhances the graph’s representational capacity, allowing the GCN to regain modeling effectiveness and receive increased weight. Simultaneously, the contribution of the temporal module diminishes and remains stable.

By comparing Figure 11 and Figure 12, notable differences can be observed. In Figure 11, the maximum CSI value approaches 1, the spatial module weight increases to nearly 1, and the temporal module weight decreases to near 0. In contrast, Figure 12 shows a maximum CSI of approximately 0.5, a peak spatial weight of around 0.8, and a minimum temporal weight of about 0.2. These discrepancies are primarily attributed to differences in the data overlapping pattern introduced by the sliding window strategy.

The EHA dataset used in Figure 11 is formed by concatenating test data from 84 consecutive days, and the input sequences span multiple days. Due to the cold-start effect of the hydraulic system, statistical discontinuities often arise at the junctions between adjacent days. When the sliding window includes such cross-day boundaries, the temporal continuity between time steps is weakened, making it more difficult for the Transformer module to extract stable temporal features. Consequently, the temporal module weight decreases significantly, and the model places greater reliance on the GCN module for modeling local structural information, resulting in an extreme increase in spatial module weight. Additionally, multiple sensor signals often exhibit synchronous fluctuations at these transition points, which strongly enhances their mutual correlation and drives the CSI value close to 1.

In contrast, the sliding window in Figure 12 is confined to a single-day operational cycle, during which system operating conditions remain relatively stable and uninterrupted by cold-start effects. As a result, both the CSI and the model’s module weights exhibit only mild fluctuations and no extreme values.

**Figure 13:**
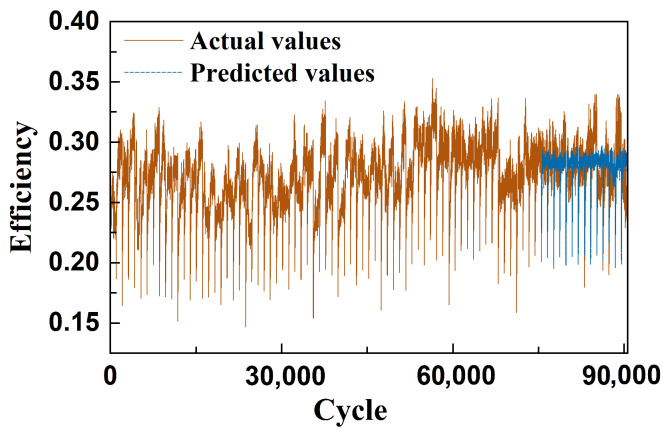
Prediction value and actual value of EHA efficiency.

Figure 13 presents the comparison between the predicted and actual efficiency of the EHA system over its full operational lifecycle. It can be observed that while the overall efficiency fluctuates significantly, it does not exhibit a clear linear downward trend but rather remains in a prolonged phase of gradual evolution. This behavior aligns with the slow upward trend of the CSI curve in Figure 11, suggesting that the degradation process of the EHA system is relatively mild and may be driven primarily by abrupt failure mechanisms, rather than cumulative efficiency loss over time.

**Figure 14:**
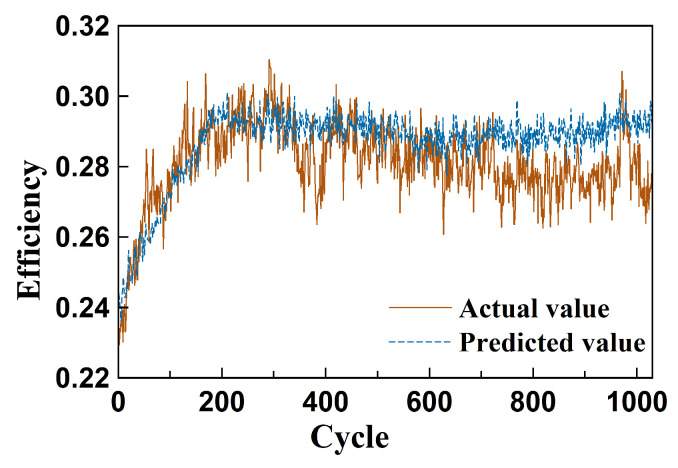
Comparison chart of predicted and actual EHA efficiency values in one day’s experiment.

Figure 14 further presents the prediction results for a single operational cycle. At the initial stage of start-up, the system efficiency is relatively low, followed by a rapid increase and eventual stabilization—demonstrating a clear transition from cold start to steady-state operation. This trend is consistent with the earlier analysis regarding high oil viscosity and elevated friction-induced energy loss during the cold-start phase. As the oil temperature rises, lubrication performance improves, leading to a rapid increase in system efficiency, which then remains stable throughout the remainder of the cycle.

Overall, the proposed model demonstrates strong fitting performance in both long-term fluctuation scenarios and short-term dynamic responses during cold start. The predicted efficiency curve accurately captures the trend and amplitude of variations, indicating that the method possesses robust modeling and generalization capabilities across different temporal scales.

It should be noted that the EHA dataset is a self-developed engineering test platform intended to validate practical applicability and interpretability under real hydraulic operating conditions. The comparative evaluation of methodological advancement is primarily conducted on the standardized C-MAPSS benchmark dataset.

## 4. Generalization Test on the C-MAPSS Dataset

### 4.1. Data Description and Preprocessing

To evaluate the applicability and generalization capability of the proposed method for RUL prediction, supplementary experiments were conducted on NASA’s C-MAPSS dataset. This dataset simulates the degradation processes of aircraft turbofan engines under various operating conditions and has become a widely used public benchmark in the field of RUL research. It comprises four subsets (FD001–FD004), each characterized by different operating scenarios and failure modes.

As summarized in Table 4, FD001 features a single operating condition and a single fault mode. FD002 includes multiple operating conditions but retains a single fault mode. FD003 consists of a single operating condition with two fault modes. FD004 represents the most complex scenario, incorporating six distinct operating conditions and two fault types, thus closely resembling real-world engineering environments.

Among the 21 sensor channels in the C-MAPSS dataset, seven channels that remain constant across the entire lifecycle are excluded following common practice in the literature. The retained 14 sensor channels are: s2, s3, s4, s7, s8, s9, s11, s12, s13, s14, s15, s17, s20, and s21.

Following the standard practice in C-MAPSS-based RUL prediction studies, the RUL values are truncated at 125 cycles to prevent excessively large early-life targets from dominating the regression scale. This setting facilitates fair comparison with existing methods.

The original dataset contains 21 sensor channels, among which 7 are constant and therefore excluded. The remaining 14 channels exhibiting dynamic behavior are retained as model inputs. Given that the dataset is a multivariate time series, a fixed-length sliding window strategy is employed to partition the data. This approach increases the number of training samples while preserving the temporal structure of the sequences.

Considering the complexity of operating conditions in FD002 and FD004, Z-score normalization after condition-based clustering is applied, following the method in [38]. This preprocessing step mitigates the impact of distributional differences across working conditions, thereby improving prediction consistency. The normalization formula is given as follows:

(21) $$x_{\mathrm{norm}}^{i}=\frac{x^{i,j}-\mu^{i,j}}{\sigma^{i,j}}$$

where $\mu^{i,j}$ and $\sigma^{i,j}$ are the mean and variance of $x^{i,j}$, respectively.

To standardize the RUL labeling and enhance training stability, this study adopts a piecewise linear degradation strategy. Specifically, the first 125 cycles are defined as the normal operation phase, during which the RUL label is fixed at 125. After this point, the label enters a linear decay phase, where it decreases linearly to zero as the number of operating cycles increases. This labeling approach emphasizes the degradation trend and simplifies the model’s learning objective.

### 4.2. Results Analysis

**Figure 15:**
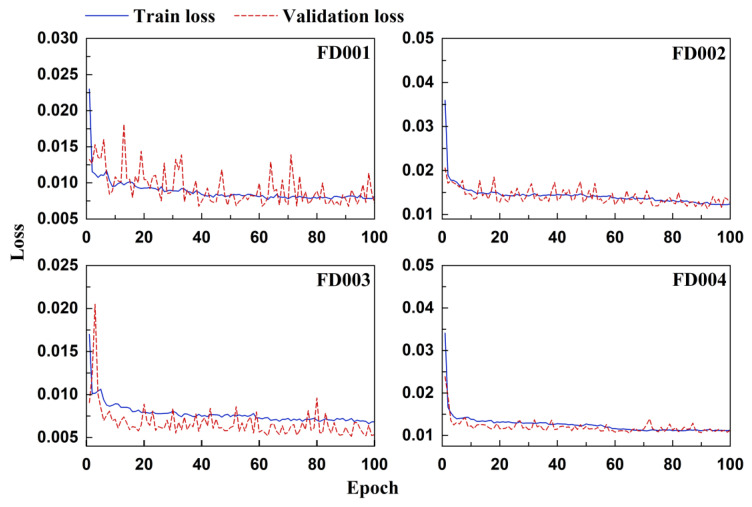
Training loss and validation loss of C-MAPSS set data.

Figure 15 illustrates the loss curves during training across the four sub-datasets of the C-MAPSS benchmark. It can be observed that both the training loss and validation loss decrease rapidly in the early stages of training and gradually converge in the later phases. The loss curves for each subset exhibit smooth convergence with minimal fluctuations, indicating that the model not only achieves good fit on the training set but also demonstrates strong generalization performance on the validation set.

Table 5 shows the ablation experimental results on each sub-dataset of C-MAPSS. The results show that pre-training significantly reduces RMSE and Score in all models and improves performance. Compared with the untrained model, the pre-trained GCN and Transformer perform better on each index. Further, after introducing dynamic weights, the adaptability of the model to different degradation stages is enhanced. Finally, the proposed pre-training + dynamic weight GCN–Transformer model (PreDyn-ST) achieves optimal results on all sub-datasets, which verifies the accuracy and robustness of the proposed method under complex working conditions.

Table 6 shows the prediction performance comparison between the proposed PreDyn-ST method and the existing mainstream methods on the C-MAPSS dataset. From the results, the proposed model achieves the best RMSE and Score on the FD002 and FD004 sub-datasets. On FD002, RMSE and Score are 2.56% and 1.28% higher than the second-best method, respectively. On FD004, the improvement is more significant, reaching 11.22% and 8.58%, respectively. In addition, although the Score of FD001 and the Score and RMSE of FD003 are slightly lower than the current best, the RMSE of FD001 remains competitive compared with other methods, demonstrating overall robustness.

It is worth emphasizing that FD004 contains the largest number of failure modes and the most complex working condition transitions, making it the most challenging subset. The superior performance on FD004 can be attributed to the joint effect of contrastive pretraining and the dynamic spatial–temporal fusion mechanism. The pretraining stage enhances the robustness of learned representations under varying operating conditions, while the adaptive weighting mechanism enables the model to adjust the relative contributions of spatial and temporal features during degradation progression. This combination is particularly beneficial in complex multi-condition and multi-mode degradation scenarios such as FD004.

It should be clarified that the physical prior graph used in the C-MAPSS experiments is not transferred from the EHA system. Instead, the same graph-construction principle is applied: system structural relationships and sensor dependencies are encoded into a graph representation according to the specific architecture of the aero-engine.

This idea is consistent with recent studies such as Cai et al. [39], where knowledge-embedded spatial–temporal GCNs were constructed for aero-engine RUL prediction by integrating component-level physical relationships into the graph structure. Therefore, the generalization demonstrated in this study does not rely on a fixed hydraulic topology but rather on the methodological framework that embeds system-level structural priors into graph learning.

Moreover, the superior performance under complex operating conditions (e.g., FD004) is not solely attributed to the physical graph prior. The dynamic weighting mechanism reduces over-reliance on spatial structural bias by adaptively balancing spatial and temporal pathways, while contrastive pretraining enhances representation smoothness across degradation stages. Together, these mechanisms enable the model to maintain robustness across systems with different physical configurations.

**Table 6:** Comparison of C-MAPSS test dataset results.

Criteria	RMSE				Score			
Dataset	FD001	FD002	FD003	FD004	FD001	FD002	FD003	FD004
CNN-LSTM [40] (2019)	14.40	27.23	14.32	26.69	290	9869	316	6594
RNN-AE [41] (2020)	13.27	19.59	19.16	22.15	228	2650	1727	2901
DARNN [42] (2021)	12.04	19.24	**10.18**	18.02	261.95	933.58	247.85	2857.44
HAGCN [21] (2021)	11.93	15.05	11.53	15.74	222.3	1144.1	240.3	1218.6
CNN–Transformer [43] (2022)	12.25	17.08	13.39	19.86	198	1575	290	1741
GAT [44] (2022)	13.82	18.52	15.07	19.02	333.14	3289.6	778.45	2262.7
GGCN [45] (2022)	11.82	17.24	15.75	20.49	**186.70**	1493.7	245.19	1371.5
ARMAGCN-GRU [12] (2023)	11.59	13.62	11.40	14.47	191.05	704.50	203.79	927.69
Res-HAS [46] (2023)	11.91	17.27	11.88	17.43	227	1199	272	2508
ATCN [47] (2024)	11.48	15.82	11.34	17.8	194.25	1210.57	249.19	1934.86
TATFA [48] (2024)	12.21	15.07	11.23	18.81	261.5	1359.7	210.2	2506.3
CTNet [49] (2025)	11.64	13.67	11.28	14.62	187	809	**187**	844
**Ours proposed**	**11.35**	**13.28**	11.13	**13.01**	240.56	**695.54**	202.39	**854.36**

**Figure 16:**
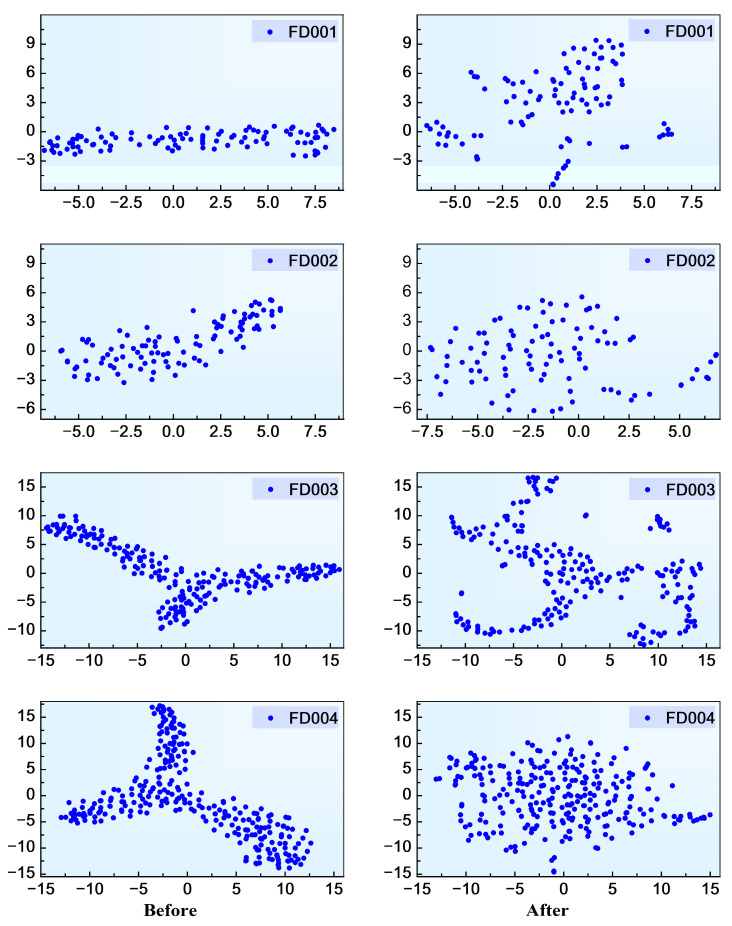
Feature distribution of C-MAPSS data before and after pre-training.

Figure 16 presents the feature distributions of each test subset in the C-MAPSS dataset before and after contrastive pretraining. Prior to pretraining, the feature points exhibit locally concentrated distributions in the two-dimensional projection space, indicating limited structural organization of the original representations. After SimCLR-based pretraining, the spatial distribution becomes more structured and progressively arranged, with clearer separation along the degradation direction. It should be emphasized that the objective of this analysis is not to demonstrate discrete class separation but to examine how contrastive learning improves the structural continuity and stage-wise distinguishability of the degradation trajectory. These observations suggest that contrastive learning facilitates a more informative embedding structure for regression-based RUL prediction, which contributes to improved convergence behavior and generalization performance in subsequent training stages.

**Figure 17:**
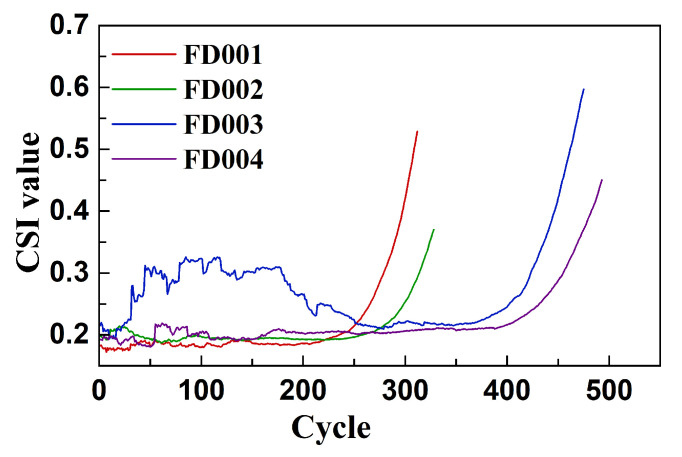
Overall CSI curve of C-MAPSS training dataset.

Figure 17 illustrates the overall trend of the Correlation Statistical Index (CSI) across the four C-MAPSS sub-datasets during the training phase. Since the training sets encompass the full degradation life cycle and share a distribution highly consistent with the test sets, the CSI curves provide a reliable representation of the evolving inter-sensor correlations throughout engine operation. As shown in the figure, the CSI curves for all datasets exhibit distinct stage-wise patterns. In the early phase of operation, the CSI values remain low, indicating weak correlations among sensor signals. In contrast, during the later degradation phase, the CSI values increase rapidly, reflecting a substantial rise in sensor coupling as the system deteriorates. This observed trend supports the assumption that the coupling strength among multiple sensors can serve as an effective indicator for identifying degradation stages. Such information is valuable for guiding the dynamic adjustment of spatiotemporal modeling strategies in response to evolving system conditions.

**Figure 18:**
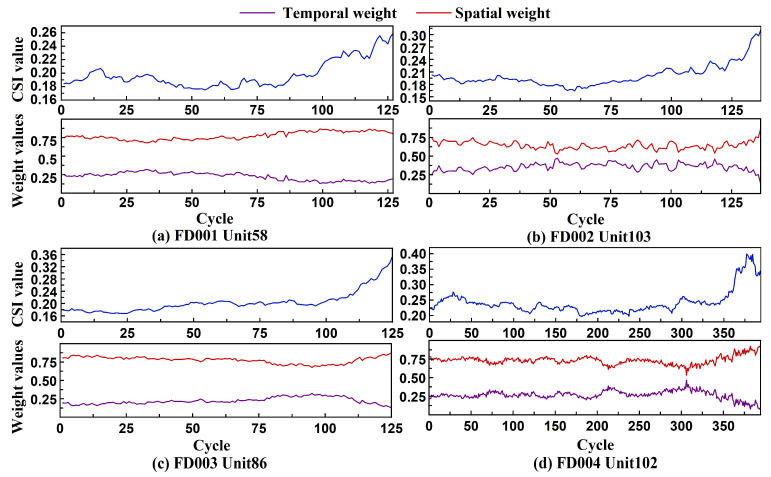
Single-engine data from the C-MAPSS testing set, CSI curve, and dynamic weight variation chart.

Figure 18 presents the CSI trajectories and corresponding dynamic weight responses of the model for a representative engine selected from each of the four C-MAPSS sub-datasets. Overall, the engine operation cycles can be broadly divided into two stages: a stable operation phase and a rapid degradation phase. During the early stable stage, the weights assigned to the temporal and spatial modules are approximately balanced. As degradation progresses, the CSI increases markedly, indicating a rise in inter-sensor correlation. In response, the model gradually shifts its weighting toward the spatial module, while the contribution of the temporal module correspondingly declines. This trend demonstrates the model’s capacity to dynamically adjust its modeling focus in accordance with changes in the temporal feature structure. Although enhanced correlation among sensors may reduce the discriminative power of local structures within the GCN, the degradation phase is characterized by non-stationary behavior within the sliding window. This disrupts long-range temporal dependencies, making it more challenging for the Transformer to capture stable temporal evolution. In contrast, the GCN is better suited for modeling localized structures and short-term interdependencies. As sensor signals become increasingly similar and exhibit stronger commonality, spatial modeling becomes more robust. The model thus adaptively increases the weight of the GCN branch, allowing it to dominate the degradation modeling process in later stages.

It is worth noting that, unlike the physically driven spatiotemporal weight transitions observed in the EHA system, the CSI trends in the C-MAPSS data—being generated from high-dimensional simulations—primarily reflect the increasing consistency of sensor responses. In this context, the introduction of a dynamic weighting mechanism enables the model to effectively identify and respond to the dominant feature structures at different stages of degradation, thereby maintaining high prediction accuracy and generalization performance in the absence of explicit physical coupling mechanisms.

**Figure 19:**
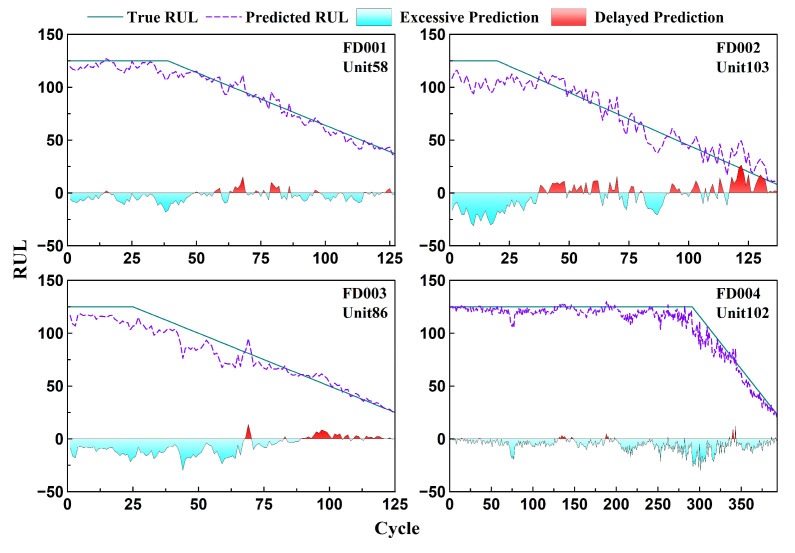
Comparison between real RUL and predicted RUL of a single engine in C-MAPSS test set.

**Figure 20:**
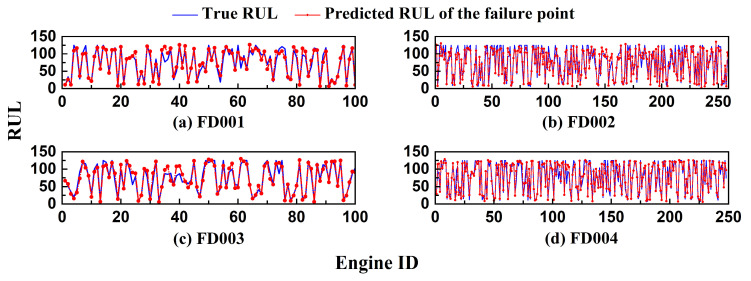
Comparison of real RUL and predicted RUL in C-MAPSS test set.

Figure 19 displays the RUL prediction curves for representative engines selected from each test set of the C-MAPSS dataset. In the early phase of operation, the model typically provides a conservative estimate close to the maximum RUL value. As the system enters the degradation phase, the predicted values progressively align with the actual downward trend of the true RUL. In most cases, the predictions fall slightly below the actual values, suggesting that the model tends to issue early warnings. This behavior aligns well with practical requirements for maintaining a safety margin in engineering applications. Although some local deviations are observed, the overall prediction trend remains accurate. Figure 20 further summarizes the predicted RUL values at the failure point for all test units. The results show that the model maintains robust performance across all four sub-datasets, with predicted values closely matching the actual RULs. These findings confirm the reliability and generalization capability of the proposed method under cross-sample conditions.

## 5. Discussion

In addition to the above validations, it is important to clarify the positioning of the proposed method relative to other methodological paradigms. Model-based fault analysis approaches rely on high-fidelity physical models and accurate parameter identification, which may be difficult to construct and calibrate for complex fluid–solid coupled systems such as EHAs under varying operational conditions. Purely data-driven methods offer strong flexibility and scalability but often lack explicit structural interpretability. In contrast, the proposed PreDyn-ST framework integrates graph-based structural modeling and contrastive representation learning with physically interpretable indicators such as CSI, providing a balance between modeling capacity and structural consistency.

Furthermore, recent domain adaptation and federated learning approaches aim to address cross-domain distribution shifts or decentralized training scenarios. These methods focus on improving generalization across different operational domains, whereas the present study concentrates on degradation modeling and dynamic feature fusion within a given system and operational environment. Therefore, the proposed framework is complementary to cross-domain adaptation strategies and is primarily designed for structured spatiotemporal degradation analysis under consistent system settings.

## 6. Conclusions

This paper proposes a spatiotemporal joint modeling framework, PreDyn-ST, for degradation prediction tasks. The overall design follows a pretraining–fine-tuning paradigm to dynamically capture temporal dependencies and spatial structural characteristics in multivariate time series (MTS) data. Specifically, contrastive learning is employed in the pretraining stage to improve representation structure and enhance feature discriminability. Subsequently, Transformer and GCN modules are utilized to model temporal dynamics and sensor topology, respectively. A learnable dynamic weighting mechanism is introduced to adaptively fuse spatiotemporal features, enabling the model to adjust feature contributions across different degradation stages.

The proposed method is validated on two representative datasets: a real-world EHA degradation test platform and the C-MAPSS benchmark dataset. In the EHA task, system efficiency is used as a physically meaningful degradation indicator, and the model behavior is analyzed in conjunction with structural characteristics and CSI visualizations to enhance interpretability. Ablation studies demonstrate the complementary roles of contrastive pretraining and adaptive spatiotemporal fusion in improving predictive performance. On the C-MAPSS dataset, the model achieves competitive and stable prediction results across all sub-datasets. Notably, it maintains robust performance on FD004, which involves multiple operating conditions and degradation modes. Compared with strong baseline methods, the proposed framework achieves improvements of 11.22% in RMSE and 8.58% in the Score metric on FD004, indicating its effectiveness in handling complex degradation scenarios.

Despite these results, several limitations remain. The current spatial modeling relies on a single-scale graph structure, which may restrict the ability to capture hierarchical or cross-level sensor interactions. In addition, while the model performs stably under gradual degradation, its sensitivity to abrupt or anomalous degradation behaviors may be further improved. Future work will investigate multi-scale graph modeling strategies and enhanced temporal representation mechanisms to strengthen robustness in non-stationary degradation environments.

## Figures and Tables

**Table 1:** Details of pre-trained model.

Parameters	Description	Value
n_length	Input sequence length	50
n_hid	Graph convolution hidden layer dimension	512
projection_dim	Output dimension of the projection layer	64
temperature	Temperature parameter for contrastive learning	0.5
num_layers	Number of layers in the encoder	2
lr	Hidden dimension	1.5 × 10^−4^
epochs	Number of training epochs	200
batch_size	Size of the sample batch	100

**Table 2:** Details of downstream task model.

Parameters	Description	Value
nfeat	Input feature dimension	16
nhid	Graph convolution hidden layer dimension	128
GCN num_layers	Number of layers in the GCN	2
num_node	Number of nodes in the graph	14
Transformer num_layers	Number of layers in the Transformer encoder	2
hidden_dim	Hidden dimension of the Transformer encoder	8
num_windows	Number of time windows	5
window_sample	Number of samples in each window	50
batch_size	Size of the sample batch	200
epochs	Number of training epochs	100
lr	Learning rate	1.5 × 10^−4^

**Table 3:** EHA dataset ablation experiment.

Method	RMSE	MAE
GCN model	2.15	0.0171
pre-trained GCN model	1.93	0.0157
Transformer model	1.93	0.0157
pre-trained Transformer model	1.89	0.0148
dynamic weight GCN–Transformer model	1.95	0.0158
PreDyn-ST	**1.86**	**0.0148**

**Table 4:** Overview of C-MAPSS test dataset.

Method	FD001	FD002	FD003	FD004
Engine units for training	100	260	100	249
Engine units for testing	100	259	100	248
Operating conditions	1	6	1	6
Fault modes	1	1	2	2
Maximum life cycle	362	378	525	543
Minimum cycles in the test set	31	21	38	19

**Table 5:** Ablation experiment of C-MAPSS test dataset.

Criteria	RMSE				Score			
Dataset	FD001	FD002	FD003	FD004	FD001	FD002	FD003	FD004
GCN model	14.13	16.51	15.95	17.41	294.31	1316.48	415.92	1579.24
Pre-trained GCN model	13.25	14.15	11.85	14.00	339.81	843.87	274.30	859.57
Transformer model	14.35	16.24	15.77	20.92	308.19	1246.18	379.00	2134.64
Pre-trained Transformer model	12.14	14.06	11.53	13.73	265.18	853.44	245.60	881.69
Dynamic weight GCN–Transformer model	13.68	16.16	15.75	20.49	251.79	1118.64	395.02	2105.86
PreDyn-ST	**11.35**	**13.28**	**11.13**	**13.01**	**240.56**	**695.54**	**202.39**	**854.36**

## Data Availability

The raw data supporting the conclusions of this article will be made available by the authors on request.
